# In Situ Study of Precipitates’ Effect on Grain Deformation Behavior and Mechanical Properties of S31254 Super Austenitic Stainless Steel

**DOI:** 10.3390/ma17112676

**Published:** 2024-06-01

**Authors:** Jinyao Ma, Huanyu Tan, Nan Dong, Jiemin Gao, Puli Wang, Zhihua Wang, Peide Han

**Affiliations:** 1 Instrumental Analysis Center, Taiyuan University of Technology, Taiyuan 030024, China; majinyao@tyut.edu.cn; 2 College of Materials Science and Engineering, Taiyuan University of Technology, Taiyuan 030024, China; tanhuanyu0287@link.tyut.edu.cn (H.T.); dongnan@tyut.edu.cn (N.D.); jmgao_stu@163.com (J.G.); wangpuli501@163.com (P.W.); 3 Institute of Applied Mechanics, College of Mechanical and Vehicle Engineering, Taiyuan University of Technology, Taiyuan 030024, China; 4 Shanxi Key Laboratory of Material Strength and Structural Impact, Taiyuan University of Technology, Taiyuan 030024, China

**Keywords:** super austenitic stainless steel, in situ tensile, grain boundary, deformation behavior

## Abstract

Grain boundary (GB) precipitation-induced cracking is a significant issue for S31254 super austenitic stainless steel during hot working. Investigating the deformation behavior based on precipitate morphology and distribution is essential. In this study, continuous smaller and intermittent larger precipitates were obtained through heat treatments at 950 °C and 1050 °C. The microstructure evolution and mechanical properties influenced by precipitates were experimentally investigated using an in situ tensile stage inside a scanning electron microscope (SEM) combined with electron backscatter diffraction (EBSD). The results showed that continuous precipitates at 950 °C had a stronger pinning effect on the GB, making grain rotation difficult and promoting slip deformation in the plastic interval. Continuous precipitates caused severe stress concentration near GB and reduced coordinated deformation ability. Additionally, the crack propagation path changed from transcrystalline to intercrystalline. Furthermore, internal precipitates were a crucial factor affecting the initial crack nucleation position. Interconnected precipitates led to an intergranular fracture tendency and severe deterioration of the material’s plasticity, as observed in fracture morphology.

## 1. Introduction

Super austenitic stainless steels (SASSs) are widely used in harsh environments such as seawater piping, chemical industries, and nuclear power tubes due to their excellent corrosion resistance and superior mechanical properties [1,2,3,4]. Among these, S31254 (Cr20-Ni18-Mo6-N0.2) has higher alloy contents (especially Mo and Cr) and significant potential for application in severely corrosive environments [5,6]. However, increasing alloy element content enhances the solid matrix solution’s strengthening effect, improving the difficulty of hot working [7,8,9]. In addition, high content of Mo and Cr leads to severe segregation and precipitation of brittle intermetallic phases like the σ phase in the Mo- and Cr-rich regions, increasing the tendency of thermal deformation cracking [10,11,12]. During hot working, the deformation temperature fluctuates significantly from 1200 °C to 900 °C [13,14,15]. The morphology, size, and distribution of precipitates vary at different temperatures, affecting deformation behavior differently. Therefore, exploring the effect of precipitation at different temperatures on deformation behavior is crucial.

Recently, many researchers have focused on precipitate formation behavior and its regulation through different aging conditions and alloying methods. Koutsoukis et al. [16] identified three intermetallic phases (χ, σ, Laves phase) and one nitride (Cr_2_N) forming in S31254 and S32654 at different temperatures. Liang et al. [17] observed that the χ phase and σ phase are the primary precipitates in SASS S31254 during aging treatment at 950 °C to 1150 °C. J. Anburaj et al. [11] studied the precipitates of forged SASS after aging for 1 h and 10 h at temperatures from 500 °C to 1000 °C. They revealed that the χ phase forms at low temperatures up to 800 °C, and the σ phase forms at temperatures above 900 °C. Li et al. [18,19] proposed that adding Ce promotes the refinement of the σ phase.

Numerous studies also focus on the deformation behavior and microstructure evolution of SASSs. Pu et al. [20] evaluated the hot workability of SASS S32654 by observing its microstructure evolution at temperatures ranging from 950 °C to 1200 °C. Adams et al. [21] found that the σ phase severely damages the mechanical properties of SASS AL-6XN. Fonda et al. [22] demonstrated the influence of coarse σ phases and void networks on the mechanical properties of SASS using 3D imaging through X-ray micro-tomography. Previous reports [23,24] indicate that precipitation significantly affects the mechanical properties of SASSs, especially the brittle phases along GB. However, studies on the interaction between precipitate morphology, distribution, and microstructure evolution remain scarce. Therefore, understanding the deformation process in depth is necessary to reveal the crack failure mechanism of SASSs during hot working.

In recent years, in situ tests have been employed to characterize microstructure evolution in real time during the deformation process [25,26,27,28,29]. However, evidence for the effect of precipitates on crack propagation in SASSs, especially under different temperature conditions, is lacking. To address this issue, this paper discusses microstructure evolution during the crack process of SASS S31254 under different aging treatments using an in situ tensile method based on SEM with an EBSD technology. The results of this study should lead to better predictions of the influence of precipitates on the mechanical properties of S31254 at different deformation temperatures and provide guidelines for regulating precipitate morphology and distribution.

## 2. Materials and Methods

### 2.1. Materials Preparation

**Figure 1:**
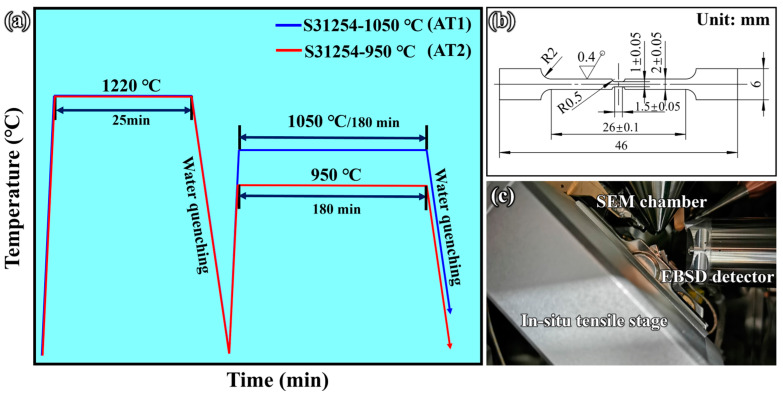
(**a**) Thermal schedule diagram of the aging process. (**b**) Dimensions of the in situ specimen. (**c**) The in situ tensile stage is embedded into the SEM chamber.

The material used in this work was provided by Technology Center, Shanxi Taiyuan Stainless Steel Co., Ltd. (Taiyuan, China), and the chemical composition is presented in Table 1. The cast ingot was manufactured by a vacuum induction melting furnace. It was firstly homogenized at 1280 °C for 10 h and then hot forged and hot rolled into a 30 mm thick steel plate. Pieces of 50 mm × 10 mm × 1 mm were cut parallel to the hot rolling direction by a wire cutting machine. The specimens were kept at 1220 °C for 25 min, followed by water quenching to room temperature. Subsequently, the specimens were kept at 1050 °C and 950 °C for 180 min separately to obtain the different distribution and morphology of precipitates, followed by water quenching to room temperature. The specimens aged at 1050 °C were labeled AT1, and those aged at 950 °C were labeled AT2. The complete thermal schedule is shown in Figure 1a.

Two types of in situ tensile specimens were prepared according to the heat treatment process of AT1 and AT2, and were machined into in situ specimens with a thickness of 0.6 mm. The detailed dimensions of the in situ specimens are shown in Figure 1b. The specimens were ground with SiC papers up to 2400 grit and then polished using W1.5 diamond paste until mirror-like smoothness was achieved. The etching process for microstructure observation was conducted using a mixed solution of 35 g FeCl_3_ + 50 mL HCl + 100 mL H_2_O.

### 2.2. Microstructure Characterization

Microstructure observation and element distribution were carried out using a TESCAN S8000G double-beam electron microscope (FIB-SEM, TESCAN, Brno, Czech Republic) with energy-dispersive spectroscopy (EDS, Ultim Max, Oxford Instruments, Oxford, UK). Crystal and local deformation evolution were characterized by electron backscatter diffraction (EBSD, Symmetry S1, Oxford Instruments, Oxford, UK) using Channel 5 software (5.12.74.0) with a step size of 0.5 μm. Identification of precipitates and morphology was carried out using transmission electron microscopy (TEM, JEM-F200, JEOL, Tokyo, Japan). TEM specimens were ground to approximately 50 μm thickness and then twin-jet electropolished at 30 V and 20 °C in 12.5 vol% perchloric acids + 87.5 vol% ethanol. The in situ tensile test was carried out by a self-developed tensile stage assembled in the SEM, and the system is shown in Figure 1c. The tensile rate during the experiment was set to 1 μm/s. A previous study provides a detailed description of the system [30].

## 3. Results

### 3.1. Microstructure Characterization

**Figure 2:**
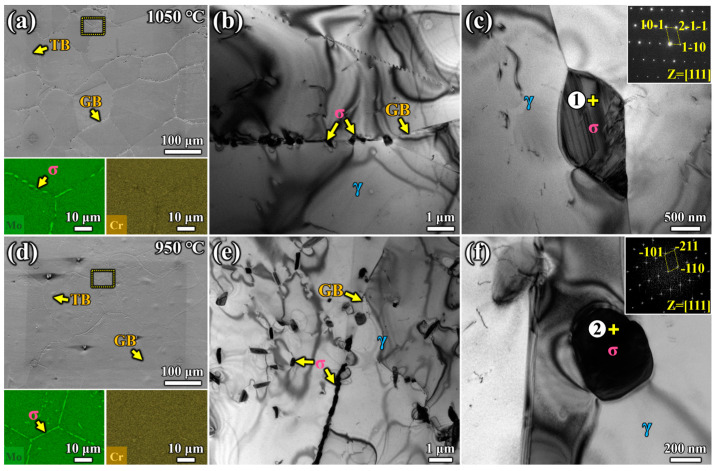
The microstructure of the different aging treated S31254: (**a**) SEM image and EDS mapping of AT1; (**b**,**c**) TEM bright-field image of AT1 and the corresponding SAED patterns of the precipitates; (**d**–**f**) SEM image and TEM images of AT2.

Figure 2a,d shows the typical micro-morphology of AT1 and AT2, respectively. Circular marks were etched by FIB in Figure 2 to maintain consistency in the observation field during the in situ experiment. Twins were observed in both AT1 and AT2 specimens, with almost no precipitates at the twin boundary (TB). The distribution and size of precipitates along the GB were also significantly different. Figure 2a,d shows the element distribution of precipitates (Mo/Cr) for AT1 and AT2 in the yellow dotted area by EDS mapping. With the increase in aging temperature from 950 °C to 1050 °C, intragranular precipitates gradually decreased, and precipitates along the GB showed an intermittent distribution. However, the size of individual precipitates increased. The precipitate kinetics of the specimen aged at 950 °C appeared significantly faster. After 180 min of aging, compared to the specimen aged at 1050 °C, almost all grain boundaries were covered with continuous σ phase when aged at 950 °C, and fine precipitates were also distributed within the grains. In particular, numerous intergranular σ phases grew into needles after aging at 950 °C. The specimen aged at 1050 °C mainly exhibited intermittent precipitates at the grain boundary, with the σ phase being wider than that in the specimen aged at 950 °C.

The types of precipitates formed in S31254 aged at 1050 °C and 950 °C were identified by TEM analysis. Figure 2 shows the TEM bright-field images and selected area electron diffraction patterns of precipitates for AT1 and AT2. As shown in Figure 2b, discontinuous precipitates were distributed along grain boundaries, with no significant intragranular precipitates in the specimen aged at 1050 °C. In the specimen aged at 950 °C, continuous precipitates appeared at the grain boundary, with many needle-like precipitates inside the grain (Figure 2e). Figure 2 indicates that precipitate distribution varied with different aging temperatures over 180 min. The precipitates were identified as the σ phase, consistent with previous studies [10,15]. The lattice parameters of two types of specimens were d_110_ = 0.62 nm (AT2 specimen), d_110_ = 0.63 nm (AT1 specimen), and d_101_ = 0.39 nm (AT2 specimen), d_101_=0.38 nm (AT1 specimen). These results were very close to those reported in the literature, with lattice parameters a = 0.882 nm and c = 0.475 nm [31,32]. The bright-field images of precipitates in Figure 2c,f were marked as 1 and 2. The mass fractions of the main compositions of precipitates are shown in Table 2.

### 3.2. Mechanical Behavior of the S31254

**Figure 3:**
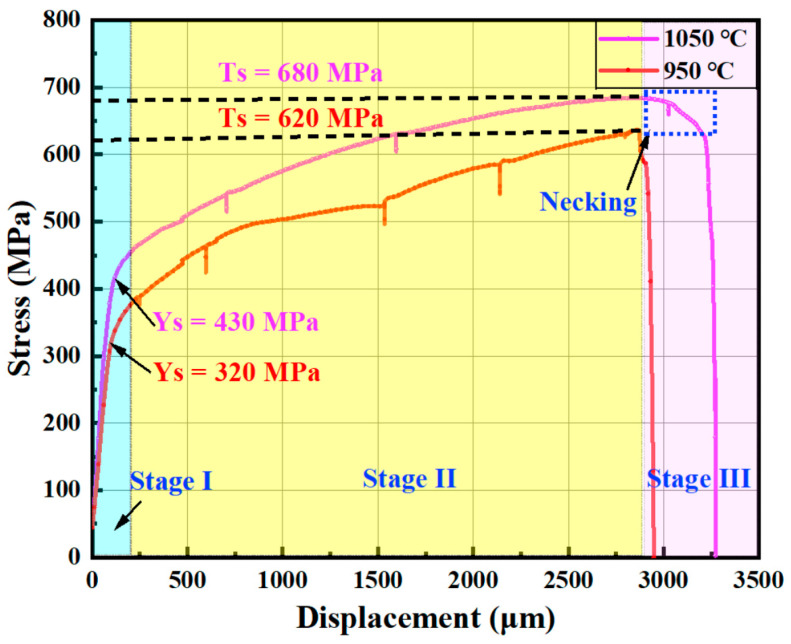
In situ tensile curves for S31254 with different aging temperatures (with unloading segment).

Figure 3 shows the tensile curves obtained with the in situ stage. An interruption during the experiment caused the unloading/relaxation segment. The relaxation effect depends on the density of mobile dislocations, back stresses, and other structural parameters [33]. However, eliminating relaxation was impossible because we needed to pause the tensile procedure for observation and EBSD scanning. In this work, there was no significant change in stress continuity due to interrupted tension; so, stress relaxation can be disregarded in the fully tensile test.

The tensile curve for the specimen aged at 950 °C differed from that of the specimen aged at 1050 °C, as shown in Figure 3. The yield strength of the specimen aged at 950 °C was 320 MPa, much lower than the 430 MPa of the specimen aged at 1050 °C. The tensile strength followed a similar trend: the specimen aged at 1050 °C had a tensile strength of 680 MPa, about 60 MPa higher than the 620 MPa of the specimen aged at 950 °C. Additionally, specimens aged at higher temperatures showed better plasticity. Specifically, after the tensile strength of the specimen aged at 1050 °C was reached, there was a slow decline in tensile stress due to the necking stage, followed by fracture. In contrast, the specimen aged at 950 °C had no obvious necking stage and fractured immediately when the applied stress reached the tensile strength. These differences in mechanical properties, such as yield strength and tensile strength, were primarily due to the different morphology and distribution of precipitates at grain boundaries. The details will be discussed later.

### 3.3. Microstructure Evolution during Tensile Deformation

**Figure 4:**
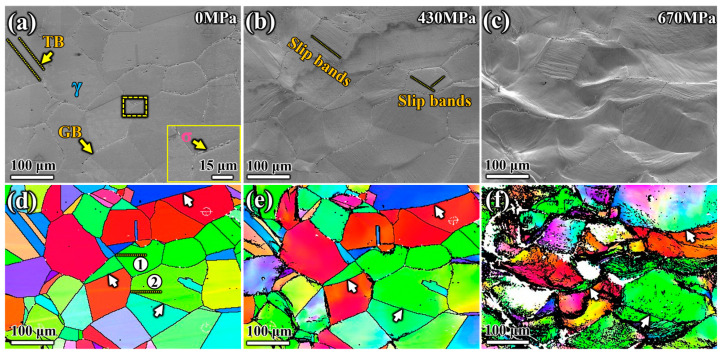
Morphology evolution images and orientation image mapping of grain surface of S31254 aged at 1050 °C for 180 min at (**a**,**d**) 0 MPa, (**b**,**e**) 430 MPa, (**c**,**f**) 670 MPa stresses during in situ tensile process.

Changes in the microstructure of the specimen aged at 1050 °C, as shown in Figure 4, were observed using in situ SEM tensile testing. In Figure 4a, the area was selected and kept within the field of view during the stretching process. Under this aging condition, the precipitates were intermittently distributed at the GB, as shown in the enlarged view in the lower right corner of Figure 4a. When the tensile load reached 430 MPa, almost all grains in the selected area exhibited plastic deformation and slip lines on their surfaces (Figure 4b). The number and direction of slip bands within each grain differed due to crystal orientation. The parallel distribution of slip bands in the same direction implied the activation of a single-slip system. A grain usually shows more slip traces, indicating that a single-slip system could not dominate the plastic deformation. As the stress continued to increase, more slip bands appeared (Figure 4c). The bending deformation of GBs and TBs with few precipitates was more significant than that of GBs with more precipitates. This phenomenon was shown in the orientation image mapping (OIM) determined by EBSD under different stresses (Figure 4d–f). White arrows marked the GBs in Figure 4d–f to observe the changes in three GBs during deformation, where no significant precipitates were distributed, according to Figure 4a,d. When the tensile stress increased to 430 MPa, significant bending deformation occurred at the three GBs without precipitates, as indicated by white arrows (Figure 4e). As the applied load increased, the bending deformation of the GBs became more apparent, as clearly shown in Figure 4f.

**Figure 5:**
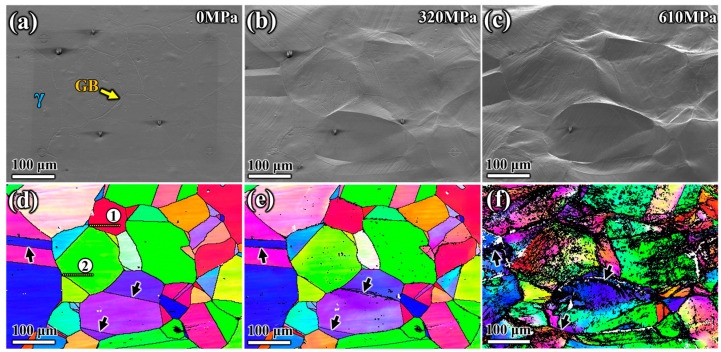
Morphology evolution images and orientation image mapping of grain surface of S31254 aged at 950 °C for 180 min at (**a**,**d**) 0 MPa, (**b**,**e**) 320 MPa, (**c**,**f**) 610 MPa stresses during in situ tensile process.

Compared to the deformation behavior of the specimen aged at 1050 °C, the precipitates along the grain boundaries were more continuous in the specimen aged at 950 °C, as shown in Figure 2d. When the specimen reached the yield stress of 320 MPa, obvious slip deformation traces appeared on the grain surfaces, as shown in Figure 5b. However, there was no obvious deformation at the grain boundaries, including the twin boundaries with almost no precipitates, as indicated by the black arrows in the OIM diagrams in Figure 5d–f. The diagrams also showed that with increased stress, all grains within the field of view elongated in the direction of the applied tensile stress. This demonstrated that the rotational deformation of grains was weaker compared to that of the specimen aged at 1050 °C. Grain deformation became prominent as the stress increased to 610 MPa. Although most twin boundaries showed no bending deformation, the migration behavior of twin boundaries during tension was more significant compared to that of the specimen aged at 1050 °C, as indicated by the black arrows in Figure 5d–f. Under external tensile stress, the positions of the TBs indicated by the black arrows shifted significantly as the stress increased, which was more evident in Figure 5f.

**Figure 6:**
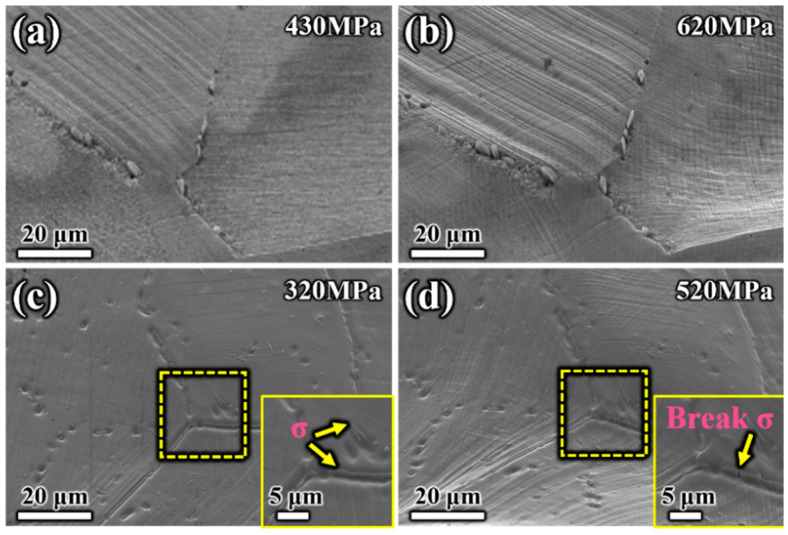
Surface morphology of S31254 with different aging temperatures for 180 min: (**a**,**b**) specimen aged at 1050 °C during tensile test at 430 MPa, 620 MPa; (**c**,**d**) specimen aged at 950 °C during tensile test at 320 MPa, 520 MPa.

Strain-induced roughness and surface morphology changes were evident in the SEM image when tilted at a 70° angle. Figure 6 shows the differences in deformation mechanisms between S31254 specimens aged at 950 °C and 1050 °C during the initial deformation stage. Many fine and dense slip lines were observed in the specimen aged at 1050 °C, with the individual heights of the slip lines being very small (Figure 6a). In the specimen aged at 950 °C, numerous fine slip lines were replaced by relatively coarse, defect-free channels, as shown in Figure 6c. Multiple-slip systems may operate within a single grain, leading to coarse slip lines and steps at the surface [34]. In addition, the higher slip steps in the specimen aged at 950 °C indicated greater local deformation and stress within the grain compared to the specimen treated at 1050 °C. Furthermore, the morphology and distribution of the slip lines indicated the uniformity of plastic deformation. Slip occurred centrally on some crystal planes, while the crystal layers between the slip bands or lines did not deform but were relatively displaced. The finer slip lines in the specimen aged at 1050 °C compared to the specimen aged at 950 °C indicated more uniform plastic deformation in the former. The different slip phenomena at 1050 °C and 950 °C can be attributed to the different distributions of precipitates after the treatments. The relatively continuous distribution of elongated precipitates along GBs may explain the higher slip steps and greater local stress and strain in the specimen aged at 950 °C. As the stress increased, some continuous precipitates along the grain boundaries were fragmented, as shown in the enlarged views of Figure 6c,d.

## 4. Discussion

### 4.1. Influence of Precipitates’ Distribution on Local Deformation Behavior

**Figure 7:**
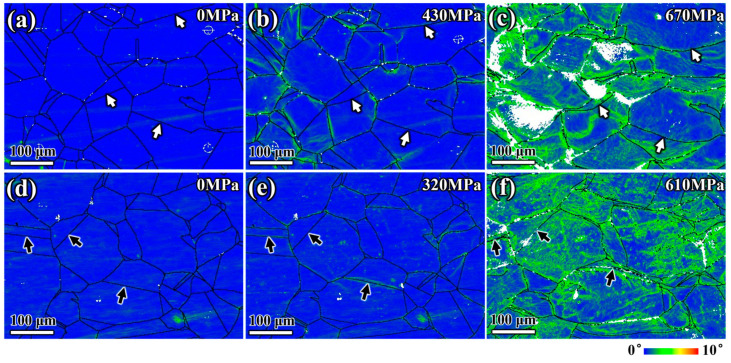
KAM maps of the surfaces for different temperature-aged specimens: (**a**–**c**) specimen aged at 1050 °C during tensile test at 0 MPa, 430 MPa, 670 MPa, respectively; (**d**–**f**) specimen aged at 950 °C during tensile test at 0 MPa, 320 MPa, 610 MPa, respectively.

During the plastic deformation stage, grains primarily coordinated the specimen’s deformation through slip formation and changes in crystal orientation. The plastic deformation behavior of grains is related to the dislocation density, especially near the GB. The kernel average misorientation (KAM) diagram measures local grain misorientation. Higher KAM values indicate more significant plastic deformation or higher defect density [35,36,37]. In the initial stage of plastic deformation, strain and dislocation density increased significantly, rapidly leading to stress concentration. For the specimen aged at 1050 °C, the KAM value at the grain boundary increased significantly when the tensile load reached 430 MPa (Figure 7b). In the specimen aged at 950 °C, compared to the specimen aged at 1050 °C, although grain boundary deformation was evident, intragranular deformation was more pronounced. This is likely due to the more significant pinning effect of continuous precipitates on the grain boundary, making grain boundary deformation difficult and weakening the ability of grain rotation and coordinated deformation. During plastic deformation, the material mainly relied on grain slip deformation. The pinning effect of precipitates on GBs can make GB deformation difficult, reducing the ability of grains to rotate and coordinate deformation, and ultimately elongating grains in the tensile direction. Additionally, the increase in precipitates consumed more Mo and Cr elements in the matrix [10,38], weakening the solid solution’s strengthening effect, reducing the yield strength of the matrix (Figure 3), and causing more significant intragranular deformation.

With continuous stretching, the stress increased. The bending deformation of some GBs in the specimen aged at 1050 °C was prominent. Meanwhile, the GB deformation gradually induced deformation in the area near the GB (Figure 7c). In the specimen aged at 950 °C, the grain boundary showed no prominent deformation characteristics compared to the intragranular deformation. The plastic deformation of the material was primarily due to intragranular deformation. Additionally, there was no more apparent bending deformation of the twin interiors and GBs, indicated by black arrows, and no precipitates, as shown in Figure 7d–f. Due to continuous precipitates along the grain boundary, the dislocation density at the grain boundary continued to increase, leading to easy dislocation accumulation (Figure 7f). The continuous increase in stress resulted in stress concentration, creating a weak link in the subsequent fracture process.

**Figure 8:**
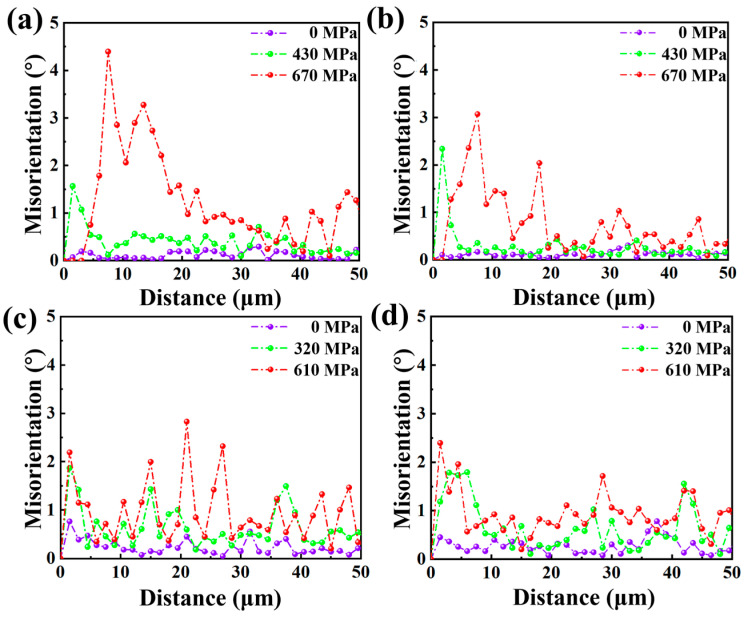
Linear misorientation distribution of a typical grains. (**a**,**b**) Lines 1,2 in Figure 4 for specimen aged at 1050 °C. (**c**,**d**) Lines 1,2 in Figure 5 for specimen aged at 950 °C.

Similarly, the misorientation profiles demonstrated that the deformation behavior differed for specimens in different aging states due to the influence of precipitation distribution along GB. Figure 8 shows that the initial misorientation angle in the four undeformed grains along the straight line did not exceed 1°. When the external load reached 430 MPa, the orientation difference of Line 1 at the grain boundary in the specimen aged at 1050 °C increased by about 1.5°, and the orientation difference of Line 2 increased by about 2.5°. As the stress continued to increase, the maximum misorientation remained near the grain boundary but gradually migrated into the crystal. However, when the external load reached 650 MPa, the orientation difference (1.5–2°) between Line 1 and Line 2 near the GB in the specimen aged at 950 °C was lower than the orientation difference (3–4.5°) in the specimen aged at 1050 °C. The misorientation was also pronounced in the region far from the GB. The misorientation could be attributed to grain plane slip deformation and rotation accumulation. Compared to the specimen aged at 950 °C, GBs with intermittent precipitates in the specimen aged at 1050 °C were easier to deform and rotate during the experiment. The phenomenon indicated that achieving a sporadic distribution of precipitates at the grain boundary through specific process control could improve the coordination of grain and GB deformation during hot working and practical applications.

### 4.2. Influence of Precipitates’ Distribution on Crack Nucleation and Propagation Behavior

**Figure 9:**
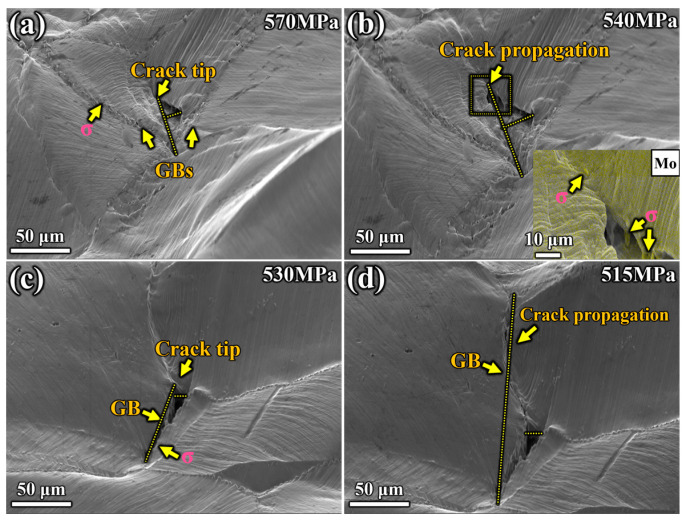
In situ microstructure observation of crack nucleation and propagation process of specimens. Specimen aged at 1050 °C at (**a**) 570 MPa, (**b**) 540 MPa. Specimen aged at 950 °C at (**c**) 530 MPa, (**d**) 515 MPa.

Through in situ experiments, it was possible to directly observe the different crack initiation and propagation modes in the two specimens. Figure 9 shows the crack nucleation and propagation process of specimens aged at different temperatures in this work. The size of the microcracks was measured along the dotted line in Figure 9. The microcracks in the specimen aged at 1050 °C had an initial length of 48.3 μm and a width of 16.1 μm. As the stretching progressed, the cracks expanded to a length of 81.0 μm and a width of 30.1 μm. In contrast, the length of the microcracks in the specimen aged at 950 °C expanded from 72.3 μm to 176.8 μm, and the width increased from 12.8 μm to 14.7 μm. In the specimen aged at 1050 °C, the crack initiated inside the austenitic grains. No obvious precipitates were observed at the crack initiation site, where severe deformation accumulation was found. Therefore, the crack initiation site was attributed to the accumulation of irreversible plastic deformation, mainly caused by the development of persistent slip bands, surface stress concentrations, and sliding displacement dislocations under high-stress conditions. In the specimen aged at 950 °C, the crack was located at the GB, where precipitates were continuously distributed. Under high-external-load conditions, the crack first occurred at the GB, where precipitates were continuously distributed in an elongated strip shape. Peeling occurred between the σ phase and the austenitic matrix under external force, and the microcracks at the front of the main crack tip remained adjacent to the σ phase. The yield strength of steel reflects its resistance to dislocation slip, as yield marks the beginning of plastic deformation accompanied by irreversible dislocation slip. Yield strength is determined by the distribution characteristics of precipitates, including their size and numerical density. Therefore, adjusting the characteristics of precipitates is the key to improving the mechanical properties of S31254.

**Figure 10:**
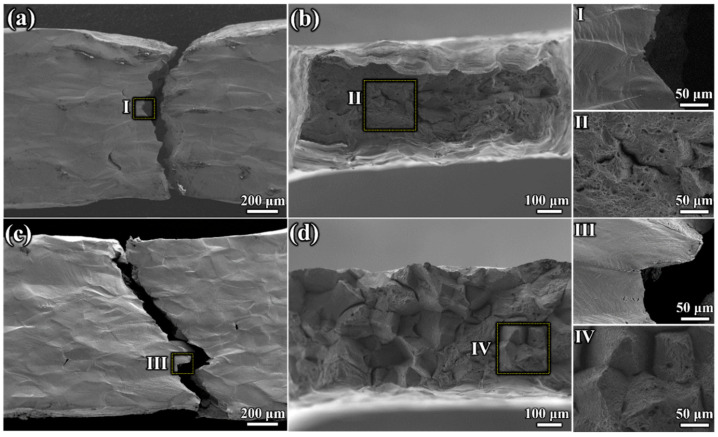
Fracture surfaces’ morphology of specimens aged at different temperatures. (**a**,**b**) Specimen aged at 1050 °C. (**c**,**d**) Specimen aged at 950 °C. (I–IV are magnified views of the rectangles in Figure 10a–d, respectively).

Figure 10 shows the fracture surfaces of the two specimens. Secondary cracks were observed on the fracture surfaces (Figure 10a,c), demonstrating a distinct mixed fracture mode of transgranular and intergranular fracture. However, the fracture in the specimen aged at 1050 °C was dominated by transgranular fracture, with apparent necking features. Additionally, dimples played a dominant role in the specimen aged at 1050 °C, while intergranular fracture was predominant in the specimen aged at 950 °C. Furthermore, a polyhedral morphology of the grains and triple junctions at the intersections of the three crystal interfaces were observed. The fracture surface exhibited many facets with different orientations, featuring small and shallow dimples.

The SEM images of the microstructure after aging at 1050 °C and 950 °C for 180 min show that the σ phases at 950 °C were almost connected in a network along the grain boundaries. The continuous distribution significantly reduced the bonding strength of grain boundaries, making them weaker than the intragranular regions and prone to intergranular fracture, resulting in lower specimen strength. The dispersed distribution of precipitates at grain boundaries at 1050 °C can pin dislocations to some extent, thereby improving the strength. At the beginning of tensile plastic deformation, dislocations inside the material accumulate uniformly at different positions. As plastic deformation progresses, certain positions develop higher stress concentrations than others due to dislocation accumulation. This leads to the formation of microcracks, which expand into macrocracks, ultimately causing the specimen to fracture. The σ phases in the specimen aged at 950 °C continuously distributed along the grain boundary, leading to dislocation accumulation, resulting in continuous stress concentration areas and serving as sites for continuous microcrack nucleation. These cracks were prone to penetration and expansion, causing intergranular damage and decreasing specimen toughness. In contrast, σ phases in the specimen aged at 1050 °C were dispersed at grain boundaries, favorably releasing the stress concentration caused by dislocation accumulation and reducing the local instability of grain boundary cracks, thereby improving specimen toughness. It is well known that the σ phase is hard and brittle, and the formation of continuous σ phases at grain boundaries can reduce strength, thereby weakening mechanical properties. Continuous σ phases are more favorable to hinder dislocation movement and generate stress concentration sites at grain boundaries. This can promote crack initiation, micropore formation, and intergranular crack propagation, thus accelerating material failure. Conversely, the dispersion of σ phases at grain boundaries is more likely to release the stress concentration, reducing the formation and connection of micro-voids, hence reducing the tendency of cracks to propagate along grain boundaries and improving both the strength and toughness. The different distributions of σ phases at grain boundaries after aging at the two temperatures were crucial in making the mechanical properties of the 1050 °C specimen better than those of the 950 °C specimen.

**Figure 11:**
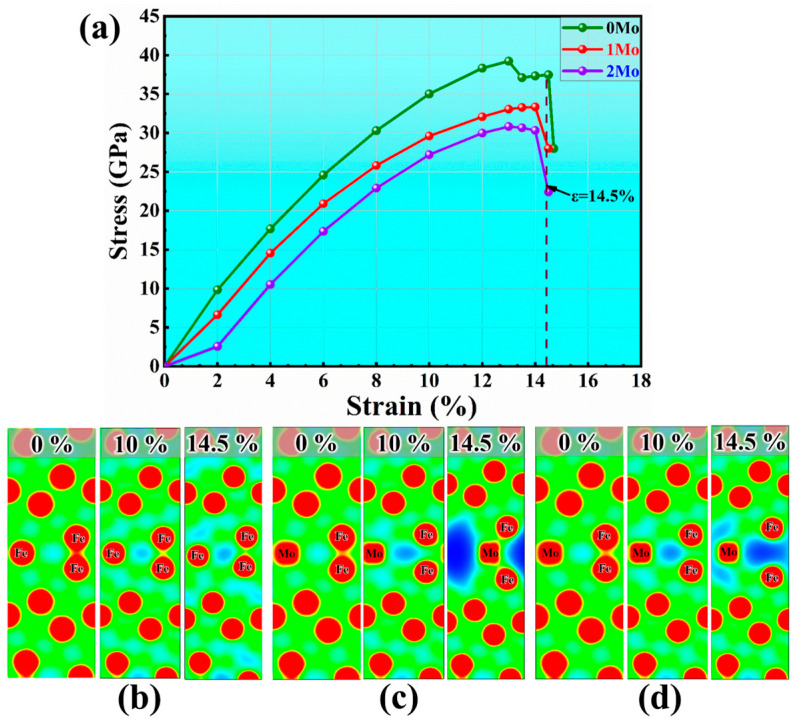
Stress–strain curve in tensile test (**a**) and charge density distribution of S31245 with a different number of Mo substitute atoms (**b**) 0Mo, (**c**) 2Mo, and (**d**) 3Mo based on first-principles.

The continuity of precipitate distribution at GBs also reflects the distribution of Mo element at GB. The more Mo atoms segregate at the grain boundary, the greater the tendency to form continuously distributed precipitates along GBs. To further understand the effects of GB strengthening and segregation element distribution, different numbers of Mo atoms at GB were investigated using the first-principle tensile test stress–strain curve and charge density distribution of S31245. The stress–strain curve showed different features for Σ3 [112] GB with 0, 1, and 2 Mo atoms (Figure 11a). The increase in tensile stress normal to the interface, with the increased number of Mo atoms at the GB, gradually decreased fracture strength and plasticity. Figure 11b–d shows the contour plots of the charge density distribution in the plane of the GBs corresponding to different strain levels. The iso-density curves with the same value of 0–0.1 e/Bohr^3^ showed that the depleted zone of charge density between Fe atoms at GB explains the weakening of interatomic bonds due to Mo segregation. This phenomenon became more significant with the increase in the number of Mo atoms at GB, corresponding to the increasingly dark blue areas in Figure 11b–d.

## 5. Conclusions

In this paper, specimens with different precipitate distribution characteristics (including size and numerical density) were obtained by aging these at different temperatures. In situ tensile tests were conducted, and the microstructure and mechanical properties during the tensile process were characterized using SEM, TEM, and EBSD. The following conclusions were drawn:

1. No apparent precipitates were observed inside the grains in the specimen aged at 1050 °C, while precipitates at GBs were distributed discontinuously. In the specimen aged at 950 °C, many acicular precipitates were inside the grains, and the precipitates exhibited an elongated strip distribution at GBs. Despite the different precipitate distributions, they were all identified as σ phase.
2. The slip lines are fine and dense, with some grains exhibiting evident multi-system slip traces during plastic deformation due to intermittent precipitates. Moreover, the grains can coordinate rotational deformation to release the stress concentration at GBs caused by external loading. Due to the elongated strip distribution of the σ phase, the atomic spacing between the slip lines becomes larger, and the pinning effect of precipitates on GBs results in more significant deformation both at GBs and inside the grains. The plastic deformation in the 1050 °C specimen is more uniform than that of the 950 °C specimen, with smaller local deformation and stress inside the grains.
3. During the tensile process of the 1050 °C specimen, a crack initiates inside the austenite grain due to the development of continuous slip bands, and stress concentration, and the accumulation of slip dislocations under high-stress conditions. In the specimen aged at 950 °C, the crack is located at the GBs, where precipitates are continuously distributed, and it propagates along GBs with continuous precipitates’ distribution under external loading. The microcracks in the front of the main crack tip are always adjacent to the σ phase.
4. The continuous distribution of precipitates at GBs is primarily related to the segregation of Mo atoms. The greater the segregation of Mo atoms, the lower the charge density between Fe atoms, weakening the interfacial bonding ability of the grain boundaries, leading to the embrittlement of GBs.
5. The intermittent distribution of granular precipitates along the GBs is the critical factor leading to a high average hardness, uniform hardness distribution, and superior strength and plasticity.

## Figures and Tables

**Table 1:** Chemical composition of the S31254 used in this study (wt.%).

Alloy	C	Cr	Ni	Mo	Si	Mn	P	S	Cu	N	Fe
S31254	0.018	19.84	18.07	6.61	0.17	1.82	0.007	0.027	1.52	0.199	Bal.

**Table 2:** Chemical composition of the precipitates in S31254 aged at 950 °C and 1050 °C for 180 min (wt.%) by TEM-EDS.

Alloy		Fe	Cr	Ni	Mo
S31254-AT1	1	37.5	22.7	9.4	13.5
S31254-AT2	2	43.5	20.1	14.1	14.5

## Data Availability

Data will be made available on request.

## References

[1] Heino S., Karlsson B. (2001). Cyclic Deformation and Fatigue Behaviour of 7Mo–0.5N Superaustenitic Stainless Steel—Stress–Strain Relations and Fatigue Life. Acta Mater..

[2] Zhang S., Jiang Z., Li H., Zhang B., Fan S., Li Z., Feng H., Zhu H. (2018). Precipitation Behavior and Phase Transformation Mechanism of Super Austenitic Stainless Steel S32654 during Isothermal Aging. Mater. Charact..

[3] Zhang S., Jiang Z., Li H., Feng H., Zhang B. (2017). Detection of Susceptibility to Intergranular Corrosion of Aged Super Austenitic Stainless Steel S32654 by a Modified Electrochemical Potentiokinetic Reactivation Method. J. Alloys Compd..

[4] Li S., Ma J., Wang J., Fan G., Li H., Jiang Z., Han P., Liang W. (2022). Impact of Boron Addition on the Hot Deformation Behavior and Microstructure Evolution of S31254. Mater. Lett..

[5] Zhang S., Li H., Jiang Z., Zhang B., Li Z., Wu J., Feng H., Zhu H., Duan F. (2020). Chloride- and Sulphate-Induced Hot Corrosion Mechanism of Super Austenitic Stainless Steel S31254 under Dry Gas Environment. Corros. Sci..

[6] Dou Y., Han S., Wang L., Wang X., Cui Z. (2020). Characterization of the Passive Properties of 254SMO Stainless Steel in Simulated Desulfurized Flue Gas Condensates by Electrochemical Analysis, XPS and ToF-SIMS. Corros. Sci..

[7] Zhang S., Li H., Jiang Z., Li Z., Wu J., Zhang B., Duan F., Feng H., Zhu H. (2020). Influence of N on Precipitation Behavior, Associated Corrosion and Mechanical Properties of Super Austenitic Stainless Steel S32654. J. Mater. Sci. Technol..

[8] Han Y., Wu H., Zhang W., Zou D., Liu G., Qiao G. (2015). Constitutive Equation and Dynamic Recrystallization Behavior of As-Cast 254SMO Super-Austenitic Stainless Steel. Mater. Des..

[9] Babu K.A., Mandal S., Athreya C.N., Shakthipriya B., Sarma V.S. (2017). Hot Deformation Characteristics and Processing Map of a Phosphorous Modified Super Austenitic Stainless Steel. Mater. Des..

[10] Zhang S., Li H., Jiang Z., Zhang B., Li Z., Wu J., Fan S., Feng H., Zhu H. (2019). Effects of Cr and Mo on Precipitation Behavior and Associated Intergranular Corrosion Susceptibility of Superaustenitic Stainless Steel S32654. Mater. Charact..

[11] Anburaj J., Nazirudeen S.S.M., Narayanan R., Anandavel B., Chandrasekar A. (2012). Ageing of Forged Superaustenitic Stainless Steel: Precipitate Phases and Mechanical Properties. Mater. Sci. Eng. A.

[12] Ebrahimi G.R., Keshmiri H., Momeni A., Mazinani M. (2011). Dynamic Recrystallization Behavior of a Superaustenitic Stainless Steel Containing 16%Cr and 25%Ni. Mater. Sci. Eng. A.

[13] Pu E., Zheng W., Xiang J., Song Z., Feng H., Zhu Y. (2014). Hot Working Characteristic of Superaustenitic Stainless Steel 254SMO. Acta Metall. Sin. (Engl. Lett.).

[14] Liu G., Han Y., Shi Z., Sun J., Zou D., Qiao G. (2014). Hot Deformation and Optimization of Process Parameters of an As-Cast 6Mo Superaustenitic Stainless Steel: A Study with Processing Map. Mater. Des..

[15] Yu J., Zhang S., Li H., Jiang Z., Feng H., Xu P., Han P. (2022). Influence Mechanism of Boron Segregation on the Microstructure Evolution and Hot Ductility of Super Austenitic Stainless Steel S32654. J. Mater. Sci. Technol..

[16] Koutsoukis T., Papadopoulou E.G., Zormalia S., Kokkonidis P., Fourlaris G. (2010). Precipitation Sequences in Cold Deformed Superaustenitic Stainless Steels. Mater. Sci. Technol..

[17] Li J., Liang W., Wu M., Zhang S., Zhang W. (2015). Microstructure Evolution in the Segregation Area of S31254 Stainless Steel Plate. Mater. Today Proc..

[18] Zhang S., Yu J., Li H., Jiang Z., Geng Y., Feng H., Zhang B., Zhu H. (2022). Refinement Mechanism of Cerium Addition on Solidification Structure and Sigma Phase of Super Austenitic Stainless Steel S32654. J. Mater. Sci. Technol..

[19] Yu J., Zhang S., Li H., Jiang Z., Feng H., Zhang B., Zhu H., Dai Y. (2023). Influence Mechanism of Cerium Addition on Precipitation Behaviour of Super Austenitic Stainless Steel S32654. J. Mater. Res. Technol..

[20] Pu E., Zheng W., Xiang J., Song Z., Li J. (2014). Hot Deformation Characteristic and Processing Map of Superaustenitic Stainless Steel S32654. Mater. Sci. Eng. A.

[21] Adams K.D., DuPont J.N., Marder A.R. (2007). The Influence of Centerline Sigma (σ) Phase on the Through-Thickness Toughness and Tensile Properties of Alloy AL-6XN. J. Mater. Eng. Perform..

[22] Fonda R.W., Lauridsen E.M., Ludwig W., Tafforeau P., Spanos G. (2007). Two-Dimensional and Three-Dimensional Analyses of Sigma Precipitates and Porosity in a Superaustenitic Stainless Steel. Met. Mater. Trans. A.

[23] Hao Y., Liu W., Li J., Nie B., Zhang W., Liu Z. (2018). Microstructural Bandings Evolution Behavior and Their Effects on Microstructure and Mechanical Property of Super-Austenitic Stainless Steel. Mater. Sci. Eng. A.

[24] Gao J., Ma J., Yang S., Guo Z., Ma J., Li H., Jiang Z., Han P. (2023). Grain Boundary Co-Segregation of B and Ce Hindering the Precipitates of S31254 Super Austenitic Stainless Steel. J. Mater. Res. Technol..

[25] Gao W., Lu J., Zhou J., Liu L., Wang J., Zhang Y., Zhang Z. (2022). Effect of Grain Size on Deformation and Fracture of Inconel718: An in-Situ SEM-EBSD-DIC Investigation. Mater. Sci. Eng. A.

[26] Bibhanshu N., Gussev M.N., Massey C.P., Field K.G. (2022). Investigation of Deformation Mechanisms in an Advanced FeCrAl Alloy Using In-Situ SEM-EBSD Testing. Mater. Sci. Eng. A.

[27] Jing W., Yongqing Z., Qinyang Z., Chao L., Wei Z., Weidong Z. (2022). In-Situ Study on Tensile Deformation and Fracture Mechanisms of Metastable β Titanium Alloy with Equiaxed Microstructure. Materials.

[28] Heard R., Siviour C.R., Dragnevski K. (2022). Investigating Iron Alloy Phase Changes Using High Temperature In Situ SEM Techniques. Materials.

[29] Ning J., Gao B., Zhou J., Chen L., Tang G., Li S. (2023). In-Situ Study on the Tensile Deformation and Fracture Mechanism of a Bimodal-Structured Mg-Gd-Y Alloy. Materials.

[30] Ma J., Lu J., Tang L., Wang J., Sang L., Zhang Y., Zhang Z. (2020). A Novel Instrument for Investigating the Dynamic Microstructure Evolution of High Temperature Service Materials up to 1150 °C in Scanning Electron Microscope. Rev. Sci. Instrum..

[31] Koutsoukis T., Redjaïmia A., Fourlaris G. (2013). Phase Transformations and Mechanical Properties in Heat Treated Superaustenitic Stainless Steels. Mater. Sci. Eng. A.

[32] Lee T.-H., Kim S.-J. (1998). Phase Identification in an Isothermally Aged Austenitic 22Cr-21Ni-6Mo-N Stainless Steel. Scr. Mater..

[33] Hariharan K., Dubey P., Jain J. (2016). Time Dependent Ductility Improvement of Stainless Steel SS 316 Using Stress Relaxation. Mater. Sci. Eng. A.

[34] Gussev M.N., Leonard K.J. (2019). In Situ SEM-EBSD Analysis of Plastic Deformation Mechanisms in Neutron-Irradiated Austenitic Steel. J. Nucl. Mater..

[35] Liu L., Huang W., Ruan M., Chen Z. (2023). Effects of Temperatures on Microstructure Evolution and Deformation Behavior of Fe–32Ni by in-Situ EBSD. Mater. Sci. Eng. A.

[36] Chen J., Lu J., Cai W., Zhang Y., Wang Y., Jiang W., Rizwan M., Zhang Z. (2023). In-Situ Study of Adjacent Grains Slip Transfer of Inconel 718 during Tensile Process at High Temperature. Int. J. Plast..

[37] Liu Q., Hansen N. (1995). Geometrically Necessary Boundaries and Incidental Dislocation Boundaries Formed during Cold Deformation. Scr. Metall. Mater..

[38] Hsieh C.-C., Wu W. (2012). Overview of Intermetallic Sigma (σ) Phase Precipitation in Stainless Steels. ISRN Metall..

